# [Retracted] Design, synthesis and preclinical evaluation of NRC-AN-019

**DOI:** 10.3892/ijo.2026.5866

**Published:** 2026-03-04

**Authors:** Kompella Amala, A.K.S. Bhujanga Rao, Bharathi Gorantla, Christopher S. Gondi, Jasti S. Rao

Int J Oncol 42: 168-178, 2013; DOI: 10.3892/ijo.2012.1697

Following the publication of the above paper, it was drawn to the Editor's attention by a concerned reader that, regarding the images of the experimental mice shown in Figs. 4A and 5A, several sets of mice were positioned in unexpectedly similar orientations, even though details of the luciferase imaging differed a little comparing between the affected data sets.

Owing to the number of duplications of data that were identified in this paper, the Editor of *International Journal of Oncology* has decided that it should be retracted from the Journal on account of a lack of confidence in the presented data. The authors were asked for an explanation to account for these concerns, but the Editorial Office did not receive a reply. The Editor apologizes to the readership for any inconvenience caused.

